# Smartphone-Based Automated Photogrammetry for Reconstruction of Residual Limb Models in Prosthetic Design

**DOI:** 10.3390/s26041251

**Published:** 2026-02-14

**Authors:** Lander De Waele, Jolien Gooijers, Dante Mantini

**Affiliations:** 1 Movement Control and Neuroplasticity Research Group, KU Leuven, 3001 Leuven, Belgium; lander.dewaele@kuleuven.be (L.D.W.); jolien.gooijers@kuleuven.be (J.G.); 2 Leuven Brain Institute (LBI), KU Leuven, 3000 Leuven, Belgium

**Keywords:** photogrammetry, residual limb, prosthetics, 3D reconstruction, deep learning segmentation, amputation, limb scanning, socket design, digital prosthetics, marker-based scaling

## Abstract

**Highlights:**

**What are the main findings?**

- A fully automated photogrammetry pipeline using only a smartphone or consumer camera achieves sub-millimeter surface accuracy and <±1% volume/perimeter error compared to CT-derived ground truth.
- Smartphone video-based photogrammetry performs robustly across diverse limb geometries, meeting all predefined clinical accuracy and repeatability thresholds for 95% of the scans.

**What are the implications of the main findings?**

- The pipeline offers a low-cost, scalable, and operator-independent alternative for prosthetic residual-limb modeling, suitable for routine clinical use and longitudinal monitoring.
- Its accessibility and automation enable deployment in resource-limited or remote care environments, supporting more equitable and data-driven prosthetic socket design.

**Abstract:**

Accurate modeling of residual limb geometry is essential for prosthetic socket design, yet current scanning techniques can be costly, operator-dependent, or impractical for repeated clinical use. This study presents a fully automated, low-cost photogrammetry workflow capable of generating metrically accurate 3D models of lower-limb residual limbs using video and still images acquired with a standard smartphone or a full-frame digital camera. The pipeline integrates adaptive frame selection, deep learning-based background removal, robust metric scaling via ArUco markers, and open-source Structure-from-Motion and Multi-View Stereo reconstruction, requiring no manual post-processing or proprietary software. Accuracy and repeatability were evaluated using four 3D-printed limb phantoms and high-resolution CT-derived meshes as ground truth. Smartphone video and full-frame camera acquisitions achieved sub-millimeter surface accuracy, volume and perimeter errors within ±1%, and high inter-session repeatability, all within clinically accepted thresholds for prosthetic socket fabrication. In contrast, smartphone still-photo reconstructions showed larger deviations and reduced stability. Acquisition time was under five minutes, and complete reconstruction required approximately 1 h and 30 min. These results demonstrate that smartphone video-based photogrammetry provides a practical, scalable, and clinically viable alternative for residual limb modeling, particularly in resource-constrained or remote care settings.

## 1. Introduction

A well-fitting prosthetic socket is crucial for restoring mobility, preventing discomfort, and ensuring long-term use of the device after lower limb amputation [1,2]. Yet producing such well-fitting sockets remains challenging due to the difficulty of accurately capturing limb geometry in a way that is fast, repeatable, and accessible [3]. As the global number of lower-limb amputees rises due to trauma, vascular disease, and diabetes, so too does the demand for more effective and scalable prosthetic solutions [4,5,6]. In clinical practice, this demand is further compounded by the need for technologies that can be deployed rapidly, require minimal operator training, and integrate smoothly into existing socket-fitting workflows that many current methods do not adequately meet.

Current state-of-the-art stump modeling approaches rely primarily on medical imaging and advanced digital acquisition techniques. Computed Tomography (CT) and Magnetic Resonance Imaging (MRI) provide highly detailed anatomical models, but they are expensive, time-consuming, and require specialized personnel and infrastructure, which limits their feasibility for repeated use in routine clinical practice [7,8]. Plaster casting remains widely adopted due to its simplicity and accessibility, yet it is labor-intensive and operator-dependent, and lacks standardization, leading to inconsistent and poorly reproducible outcomes [9,10]. Optical 3D scanning systems, including laser-based (e.g., Faro Freestyle, Creaform HandySCAN) and structured-light devices (e.g., Artec Eva, Creaform Go!SCAN), offer a fast, accurate, non-invasive way to capture stump geometry and integrate well with digital Computer-Aided Design and Computer-Aided Manufacturing workflows. However, dedicated scanners remain relatively expensive, with typical costs in the range of €10,000 to €25,000 or higher. Commercial handheld structured-light scanners frequently used in prosthetics and orthotics, such as medical-grade systems reviewed by Dickinson et al. and Silva et al. [11,12], typically achieve sub-millimeter geometric accuracy (approximately 0.1–0.5 mm) under controlled conditions. While this level of accuracy is sufficient for residual limb shape capture, reliable acquisition generally requires the subject to remain stationary during scanning and depends on operator expertise, which challenges their integration into clinical workflows [11,12,13]. Notably, laser-based scanners are generally not adopted for direct residual limb scanning in clinical prosthetics practice. Dickinson et al. [11] explicitly selected structured-light systems over laser-based alternatives due to considerations of patient safety, robustness on compliant soft tissue, and practical clinical usability.

Given these challenges, there is a clear need for stump modeling solutions that balance anatomical accuracy with practical feasibility. An ideal technique should capture residual limb geometry and be non-invasive, cost-effective, and suitable for repeated use. A truly effective solution should detect subtle changes in shape and volume over time, support regular monitoring, and integrate easily into digital prosthetic workflows. During the early post-operative and rehabilitation phases, residual limb volume can change substantially, with reported long-term reductions of approximately 17–35% over several months. Even after limb maturation (≥12–18 months post-amputation), clinically relevant diurnal volume fluctuations of approximately −1.5% to +2.0% have been reported [14,15]. Because these short-term fluctuations are sufficient to alter socket fit, interface pressures, and user comfort, measurement methods intended for ongoing monitoring must achieve volume accuracy below 1% to reliably detect meaningful changes and prevent fit-related complications. These features would enable both prosthetists and users to anticipate fit-related issues before they compromise comfort or mobility, ultimately improving rehabilitation outcomes and long-term prosthesis use [15,16].

Photogrammetry enables the reconstruction of 3D surfaces from overlapping 2D images using algorithms like Structure-from-Motion (SfM) and Multi-View Stereo (MVS) [17]. This technique only requires a standard digital camera to capture the residual limb, making it a contactless, affordable, and highly portable approach [18,19]. This makes it attractive for prosthetic applications; however, its clinical accuracy and integration into routine workflows still require further validation.

Several early feasibility studies have demonstrated photogrammetry’s potential in prosthetics and orthotics. Hernandez and Lemaire [20] introduced a smartphone-based technique for digitizing socket interiors, achieving an average reconstruction error of 2.6 ± 2.0 mm. Similarly, Cullen et al. [21] reported that a low-cost smartphone workflow could digitize positive socket and limb casts with 99.65% and 99.13% accuracy in surface area and volume, respectively. In a complementary investigation, Walters et al. [22] evaluated commercially available smartphone scanning applications for in vivo residual limb assessment, using a structured-light Artec EVA scanner as the reference standard. For the two best-performing applications (Polycam and Luma), volume measurements demonstrated mean volume differences of −2.9% (Polycam) and −1.0% (Luma) and geometric surface comparisons yielded root mean square error (RMSE) of 1.99 mm (Polycam) and 2.36 mm (Luma). A recent comparative study showed that both photo-based and video-based photogrammetry produce reliable measurements for facial asymmetry, although video-based photogrammetry yields noticeably smoother models [23].

Researchers have also explored combining photogrammetry with rapid fabrication. Ismail et al. [18] used a digital camera to model a transradial residual limb and successfully 3D-printed a socket, demonstrating substantial reductions in cost and production time.

Collectively, these studies illustrate that photogrammetry can produce usable models and streamline socket design. However, photogrammetry as a technique is inherently sensitive to acquisition conditions, including lighting, surface texture, and reflectance, and typically involves non-negligible processing times compared with direct 3D scanning methods. In addition, these previous studies rely on commercial photogrammetry software (e.g., Autodesk ReCap, Agisoft Metashape), require manual scaling and mesh cleaning, and lack full automation, factors that limit reproducibility and integration into clinical workflows. Moreover, critical clinical validation is lacking, both in assessing whether reconstruction errors fall within clinically relevant thresholds and in benchmarking results against a definitive ground-truth reference such as CT [21,24,25]. Without such validation, it remains unclear whether they can support routine clinical decision-making.

This highlights a key methodological gap: the absence of a fully automated and clinically validated photogrammetry pipeline that provides quantitative benchmarking against high-resolution ground-truth limb models based on socket-fitting performance criteria. Addressing this gap would advance the state of the art by offering clinically comparable geometric accuracy within a faster, more practical, and less operator-dependent workflow, which is essential for routine longitudinal monitoring and for scaling stump-modelling methods across diverse clinical environments.

To address this gap, we present a fully automated photogrammetry pipeline for modeling residual lower limbs using images and videos captured with both a standard smartphone and a high-resolution full-frame camera. Unlike previous approaches, our pipeline requires no manual intervention and uses no commercial software. We validated its accuracy by comparing photogrammetric outputs to CT-derived models of 3D-printed residual limbs, focusing on shape and volume metrics that are critical to socket design.

## 2. Materials and Methods

**Figure 1:**
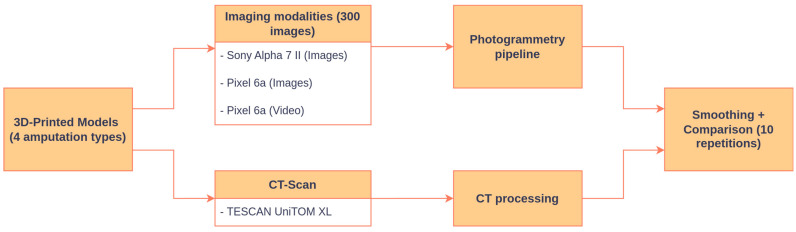
Schematic overview of the experimental pipeline for photogrammetry and CT-based model comparison.

This subsection provides a general overview of the experimental workflow used in this study; detailed descriptions of each component are provided in the dedicated sections that follow. An overview of the experimental workflow in this study is shown in Figure 1. Four 3D-printed anatomical models, each representing a different type of lower-limb amputation, were used as ground-truth objects. These models were derived from a publicly available database of residual-limb anatomies, i.e., real residual limbs from actual patients [26]. Each model was digitized using two independent modalities: photogrammetry and CT scanning, enabling controlled, high-precision comparisons. The overall aim of the workflow was to evaluate whether a fully automated photogrammetry pipeline, based on consumer-grade devices, could reproduce CT-derived geometry within clinically accepted tolerances for global geometric metrics and prosthetic-relevant shape descriptors.

For photogrammetry, images were captured using a high-resolution full-frame camera (Sony Alpha 7 II, Sony Group Corporation, Tokyo, Japan), and both images and videos were acquired with a standard smartphone (Google Pixel 6a, Google LLC, Mountain View, CA, USA). A customized SfM and MVS pipeline was used to reconstruct 3D models from still images and video frames. CT scans of the 3D-printed models served as high-accuracy ground-truth references, allowing assessment of both geometric accuracy and clinical relevance of the reconstructions.

All processing steps were executed on a high-performance workstation (Intel Core i9-10980XE, 256 GB RAM, NVIDIA Quadro RTX 4000, CUDA 12.4), using Python 3.12.3 and open-source photogrammetry tools. The following sections describe (1) the photogrammetry pipeline, (2) CT-based ground-truth generation, (3) the acquisition protocol, and (4) the accuracy and repeatability evaluation framework.

Detailed implementation parameters, algorithmic settings, and extended validation tables are provided in the Supplementary Materials to maintain readability of the main manuscript while ensuring full methodological transparency.

### 2.1. Photogrammetry Pipeline

**Figure 2:**
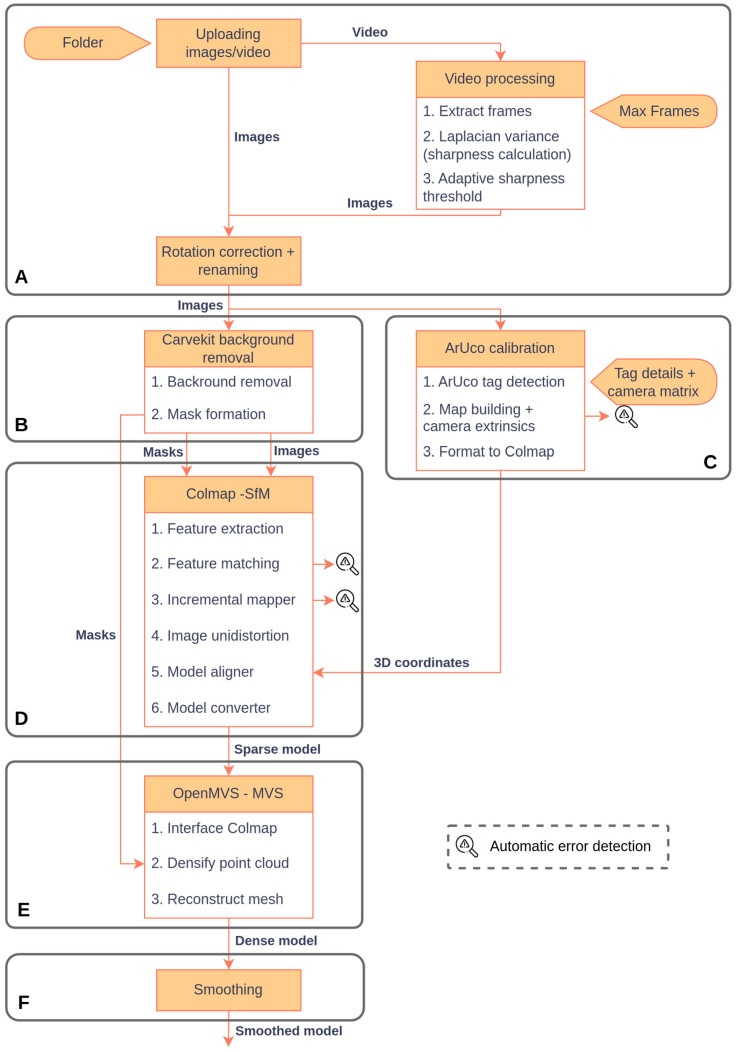
Photogrammetry reconstruction pipeline from raw image/video input to scaled 3D model generation. (**A**) Image/video acquisition and preprocessing; (**B**) Background removal; (**C**) ArUco calibration; (**D**) Structure-from-Motion; (**E**) Multi-View Stereo; (**F**) Smoothing.

**Figure 3:**
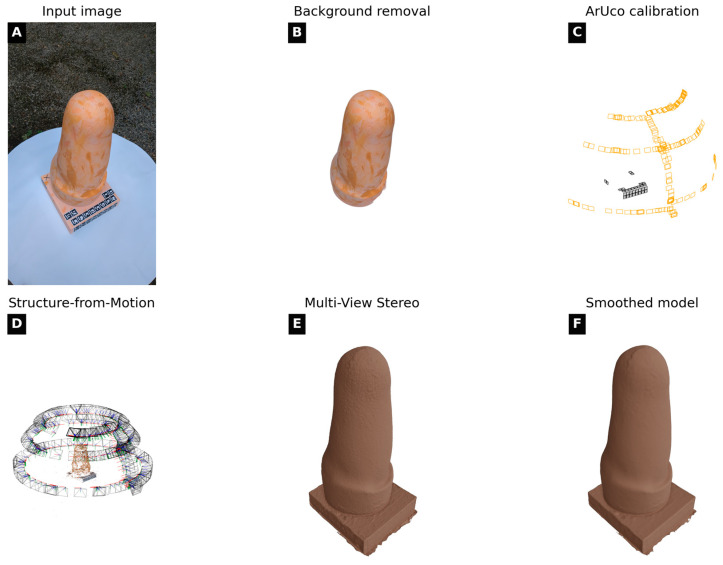
Visualization of the photogrammetry reconstruction steps from raw image to smoothed dense 3D model. (**A**) Image/video acquisition and preprocessing; (**B**) Background removal; (**C**) ArUco calibration; (**D**) Structure-from-Motion; (**E**) Multi-View Stereo; (**F**) Smoothing.

The photogrammetry pipeline is entirely automated and implemented through a Linux-based Bash script. It consists of six sequential stages (Figure 2 and Figure 3), managing preprocessing, segmentation, scaling, SfM, dense reconstruction, and smoothing without manual intervention. All photogrammetry tools used in this study are open-source. COLMAP (v3.11.1), OpenMVS (v2.3.0), CarveKit (v4.1.2), and PyMeshLab (v2023.12) are publicly available via their respective repositories.

#### 2.1.1. Image/Video Acquisition and Preprocessing

To standardize input across image and video datasets, preprocessing first identifies whether the input folder contains still photographs or smartphone video recordings. For video input, frames are extracted using FFmpeg (v6.1.1), after which a sharpness-based adaptive frame selection is applied. Sharpness is estimated from the variance of the Laplacian, and a moving-window threshold ensures that only locally sharp frames are retained, reducing motion blur effects inherent in handheld acquisition.

All selected images are automatically renamed using a standardized scheme and reoriented using EXIF metadata to maintain consistent geometry. This ensures that only corrected, top-left–aligned images enter the photogrammetry pipeline.

#### 2.1.2. Background Removal

To isolate the object of interest, background removal is performed using CarveKit (v4.1.2) (TRACER-B7 model, CUDA-accelerated) [27,28]. The resulting alpha masks are thresholded at 50% to obtain binary segmentation. This automated segmentation step increases robustness under uncontrolled environmental conditions and allows for relative motion of the model within the environment.

#### 2.1.3. ArUco Calibration

Augmented Reality University of Cordoba (ArUco) markers [29] are used to achieve metric scaling and extrinsic camera parameters initialization, which are crucial for obtaining geometrically reliable reconstructions from consumer-grade devices. The adapted pytagmapper [30] workflow identifies marker corners with subpixel refinement, estimates per-image camera poses via a two-stage Perspective-n-Point procedure, and then constructs a globally consistent map using joint optimization over all detected tags and cameras. This ensures scale-accurate reconstructions without manual interventions.

Extrinsic camera parameters are exported into compatible trajectories, enabling accurate scaling of the sparse model produced by the SfM stage. This ensures a metrically scaled final model with measurement traceability to the ArUco tag reference.

#### 2.1.4. Structure-from-Motion

SfM estimates camera poses and a sparse 3D point cloud from overlapping images. In the processing pipeline, it is performed with COLMAP (v3.11.1) [31,32] inside a standardized Docker container. Feature extraction used GPU-accelerated scale-invariant feature transform keypoint detector and foreground masks (see Section 2.1.2) to restrict features to the limb model. A single calibrated PINHOLE camera model is used, with the same intrinsic camera parameters used for ArUco calibration.

Exhaustive feature matching establishes correspondences between all images, after which incremental mapping produces a sparse 3D model using bundle adjustment to minimize reprojection error. If mapping fails (e.g., poor initialization), the pipeline automatically retries using the best initial image pair extracted from COLMAP logs. Sparse reconstructions are aligned to the ArUco reference frame using COLMAP’s rigid alignment tool and exported for dense reconstruction.

#### 2.1.5. Multi-View Stereo

MVS then refines the sparse point cloud into a dense 3D surface. This is performed in the processing pipeline using OpenMVS (v2.3.0) [33]. The COLMAP model is converted using InterfaceCOLMAP, and CarveKit masks are supplied to restrict densification to the limb model. DensifyPointCloud produces a high-resolution point cloud, followed by visibility filtering and mesh reconstruction via ReconstructMesh. For the high-resolution full-frame setup, a controlled downscaling during densification reduces runtime while preserving geometric fidelity.

#### 2.1.6. Smoothing

Meshes are smoothed using PyMeshLab’s (v2023.12) [34] surface-preserving Laplacian filter (20 iterations, max normal angle 90°) to reduce noise while maintaining anatomical geometry. The same smoothing parameters are applied to CT meshes to ensure consistent post-processing.

### 2.2. Validation

To evaluate the accuracy, repeatability, and clinical applicability of the proposed workflow, a comprehensive validation protocol was conducted using 3D-printed residual limb phantoms and CT-derived ground-truth meshes. The following subsections provide an overview of the key steps, while detailed descriptions and parameter choices are provided in the validation section of the Supplementary Materials.

#### 2.2.1. Limb Model Preparation

**Figure 4:**
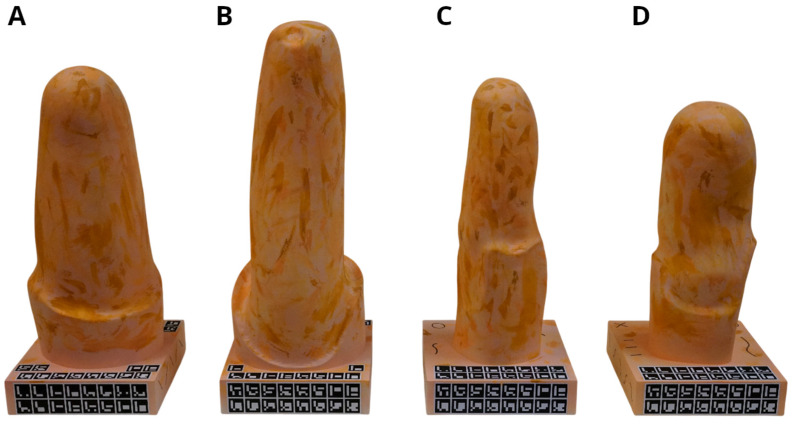
3D-printed phantoms of lower limb residual limbs with ArUco tags and skin-colored texture: (**A**) TF Aqua, (**B**) TF Ischial, (**C**) TT Conical, (**D**) TT Cylindrical.

Two transfemoral (TF) and two transtibial (TT) amputation models were selected (TF Aqua, TF Ischial, TT Conical, and TT Cylindrical) (Figure 4), based on the classification by Cutti et al. [35], which identifies these four shapes as representative of distinctly different residual limb and socket geometries. Models were downloaded from a Dryad repository [26] and prepared for 3D printing by adding a flat base and closing small mesh holes. The models were then fabricated in white SLA resin via MakerVerse (Berlin, Germany).

Because the raw 3D prints were uniformly white and lacked surface texture, the models were painted using several different water-based acrylic skin-like color tones to introduce controlled variations in shading and low-level features. While these patterns do not replicate real skin texture, the use of multiple naturalistic tones ensures the presence of the surface features required for SfM reconstruction and reflects the type of contrast typically observed on real residual limbs.

Thirty-two ArUco markers (2 cm) were attached to the model base to enable metric scaling. Additional black lines were drawn on the base to further support feature extraction in SfM.

#### 2.2.2. Ground-Truth Generation

Although only external surface geometry was required in this study, CT was selected as the reference modality to provide a geometrically traceable and modality-independent ground truth. Unlike optical 3D scanners, CT-based reconstruction is not influenced by surface texture, reflectance, lighting, or line-of-sight effects, which can introduce systematic bias when used as a reference for photogrammetric validation. Importantly, the CT volumes were used exclusively to extract the outer shell of the printed specimens; internal structures were not considered. This ensures direct comparability with optical surface reconstructions while leveraging the known isotropic resolution and well-defined uncertainty of the CT acquisition.

High-resolution CT volumes were acquired using a TESCAN UniTOM XL at the KU Leuven XCT Core Facility and reconstructed in Penthera™ with isotropic 100 µm voxels. This resolution defines the intrinsic spatial uncertainty of the CT-derived reference models and therefore represents a lower bound on measurable surface deviations. A custom Python pipeline processed the CT data in overlapping slabs to avoid boundary artifacts. A global Otsu threshold was applied to isolate the printed material, after which the outer shell was extracted using binary propagation seeded at the slab boundaries. A signed distance field was then computed, smoothed, and converted into a mesh using marching cubes. After concatenating and welding the per-slab meshes, Taubin smoothing and the same surface-preserving smoothing techniques as for the photogrammetry meshes were applied.

#### 2.2.3. Image Acquisition Setup

Image acquisition for this study was performed using two camera systems: a Google Pixel 6a smartphone (used for Pixel photo and Pixel video modalities) and a Sony Alpha 7 II with a 50 mm prime lens, serving as the high-resolution full-frame camera. In total, 120 acquisitions were collected by a single trained operator (10 repeats × 4 phantom models × 3 imaging modalities).

All captures were performed outdoors under natural daylight, using fixed manual focus and three viewpoint trajectories (low-angle, eye-level, high-angle) to ensure full 360° coverage. All acquisitions were performed handheld, without any additional mechanical stabilization (e.g., tripods, gimbals, or rigs), and without the use of any auxiliary or artificial lighting. Outdoor acquisition under natural daylight was selected to provide uniform, diffuse illumination and to minimize hard shadows. For the smartphone, identical acquisition parameters were used for both still-image and video capture. Acquisition parameters are summarized in Table 1. These settings were selected empirically through preliminary testing, during which multiple combinations of focus, distance, lighting, and exposure were compared to identify the configurations that produced the most stable and feature-rich reconstructions for our pipeline. Because no standardized acquisition protocol exists for residual-limb photogrammetry, parameters were optimized through iterative trial-and-error rather than derived from previous studies. Camera intrinsic parameters for both devices were calibrated using OpenCV with subpixel refinement and nonlinear least-squares optimization based on 50 checkerboard images per device.

#### 2.2.4. Performance Assessment

Computation times for each processing step and imaging modality were also recorded. These values are reported descriptively only, without statistical comparison, as processing time depends strongly on hardware specifications, memory bandwidth, background processes, and GPU availability.

Based on the work of Cutti et al. [35], a clinically meaningful region of interest (ROI) was defined from the distal limb end to the established anatomical landmarks: the Mid-Patellar Tendon (MPT) for TT models, and the midpoint between the origin of the Adductor Longus and the Ischial Ramus (BAR) for TF models. All models were aligned to the CT reference using a two-stage registration: coarse alignment with fast point feature histograms and random sample consensus, followed by multi-scale point-to-plane iterative-closest-point [36].

Surface accuracy was assessed using clinically established metrics: mean radial error (MRE), which captures global systematic bias; root mean square error (RMSE) and interquartile range (IQR), which quantify overall surface variability; Hausdorff distance (maximum surface deviation), which highlights local worst-case geometric discrepancies relevant for identifying potential pressure-sensitive regions; and mean angular error (MAE), which reflects surface normal noise [13,35]. Clinical thresholds required |MRE| ≤ 0.25 mm, IQR ≤ 0.4 mm, RMSE < 1 mm, Hausdorff ≤ 1.8 mm, and MAE < 4° [13,35]. Orthotics & Prosthetics metrics included relative error in cross-sectional perimeter (10 axial slices) and ROI volume (VErel). Bias and minimal detectable change (MDC), quantified accuracy and repeatability, with clinical limits of ±1% bias and MDC < 3.5%. These clinical thresholds were adopted from previously validated studies establishing geometric accuracy requirements for prosthetic socket design [11,35,37].

Reconstruction accuracy metrics were summarized descriptively and evaluated against these predefined clinical thresholds. Because the purpose of the analysis was to determine whether each reconstruction modality independently met clinically acceptable accuracy criteria, rather than to test for statistical differences between modalities, and because the pool of available smartphones and full-frame cameras is highly heterogeneous and rapidly evolving, making direct comparative claims between modalities not stable or generalizable, no inferential comparisons between modalities were performed. Although descriptive differences can be observed, modality selection is guided by threshold compliance and practical feasibility rather than by statistical superiority.

Repeatability was defined as the stability of the reconstruction for the same limb model imaged repeatedly using the same modality. It was assessed by selecting a medoid mesh from each set of 10 repeats and computing signed point-to-surface distances from this reference to all repeats. For each vertex of the medoid, local measurement variability was quantified using per-vertex standard deviation (SD), IQR, and the 95th percentile (P95), providing spatially resolved repeatability metrics. In addition to these local measures, all signed distances across all vertices and repeats were pooled into a single distribution, from which the global SD was computed. This global/pooled SD was then used to derive the MDC as the global repeatability metric.

Reproducibility was defined as the consistency of reconstruction performance across different limb geometries within the same imaging modality. To evaluate this, a one-way ANOVA with amputation type as the factor (4 levels; α = 0.05) was performed separately on VErel and relative perimeter error. The purpose of this analysis was to determine whether reconstruction performance depended on the underlying limb geometry, not to identify which individual geometries differed from each other. Because we did not intend to perform post hoc pairwise comparisons between the four models, no correction for multiple comparisons was required. This choice allowed us to assess global between-model robustness of the reconstruction pipeline without reducing statistical power in this exploratory setting.

## 3. Results

### 3.1. General Performance

**Figure 5:**
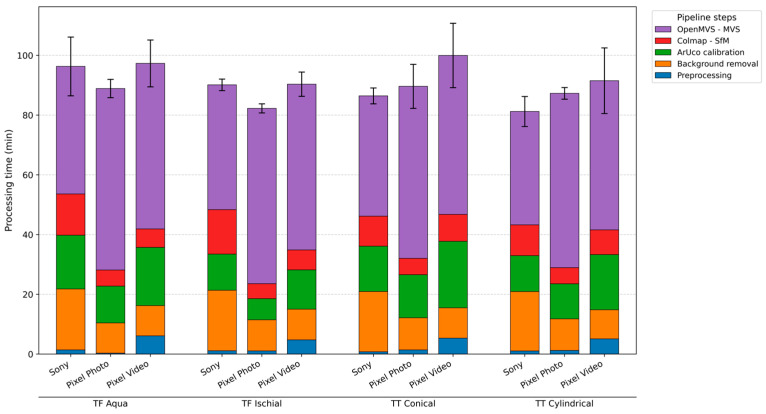
Processing time of each pipeline step, per modality and limb model. The time is expressed in seconds, separately for the following processing steps: Preprocessing, Background removal, ArUco calibration, Structure-from-Motion, and Multi-View Stereo. Error bars represent the standard deviation (SD).

Figure 5 summarizes the total processing time of the photogrammetry pipeline, which required approximately 1 h and 30 min per reconstruction, with variability of about ±15 min. The OpenMVS dense reconstruction (MVS) step was consistently the most time-consuming component, typically accounting for more than 50% of the total runtime across all datasets. Although the three reconstruction modalities showed broadly comparable overall processing times, their distribution across steps differed notably. For the Sony-based reconstructions, the higher native image resolution resulted in proportionally longer background removal and SfM steps. However, because the pipeline applies controlled downscaling before the MVS stage for high-resolution inputs, the dense point cloud generation for the Sony data was approximately 15 min faster, ultimately yielding similar total runtimes across modalities.

On average, video acquisition required only 2 min, markedly shorter than photo-based acquisitions, which required 5–10 min due to repositioning and manual shuttering.

### 3.2. Reconstruction Accuracy

**Figure 6:**
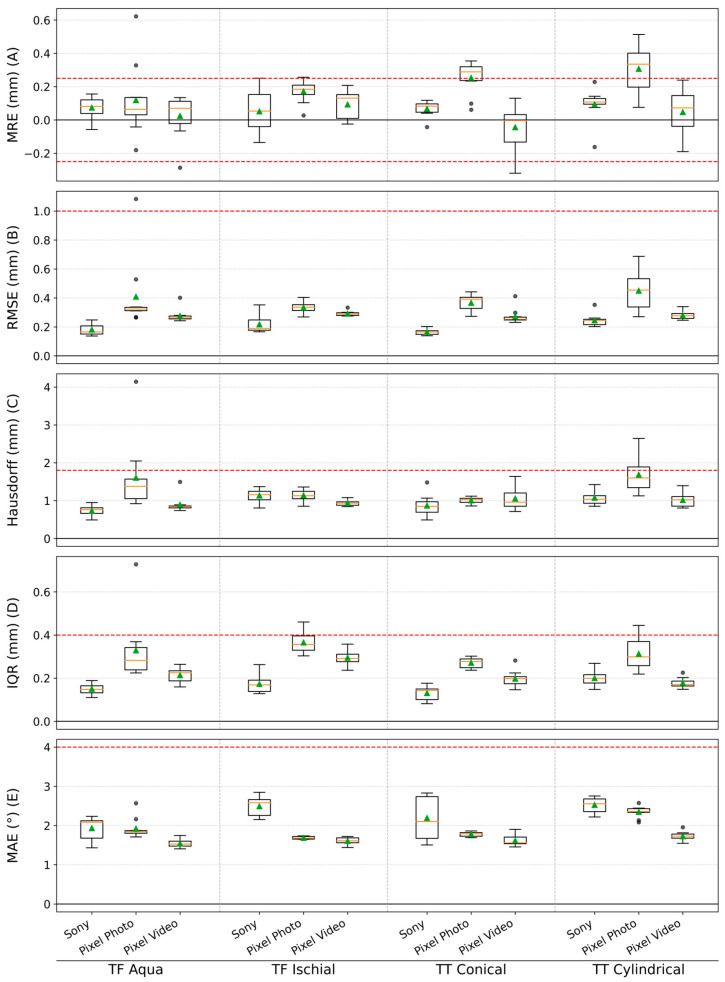
Box plot summary of global geometric and angular accuracy metrics for photogrammetric reconstructions across imaging modalities and limb models. Metrics include: (**A**) mean radial error (MRE); (**B**) root mean square error (RMSE); (**C**) Hausdorff distance; (**D**) interquartile range (IQR); and (**E**) mean angular error (MAE). Box plots represent median values with interquartile ranges across ten repeated acquisitions; green triangles indicate mean values. The whiskers extend to the most extreme data points that lie within 1.5 × IQR of the lower and upper quartiles, and individual dots represent outliers beyond this range. Red dashed lines denote clinically acceptable tolerance thresholds for each metric [35].

Figure 6 presents the global accuracy metrics (MRE, RMSE, Hausdorff distance, IQR, and MAE) for the photogrammetry reconstructions obtained with different camera setups and targets.

Across all metrics, the three modalities exhibited distinct patterns. In particular, the Pixel Photo modality showed larger reconstruction errors and a greater number of outliers at a descriptive level. As stated in the Methods, these observations are not based on inferential comparisons between modalities but serve only to contextualize each modality’s performance relative to the predefined clinical thresholds.

The red dashed lines indicate predefined clinically relevant accuracy thresholds. The Pixel Photo modality exceeded the predefined accuracy thresholds more frequently than the other modalities in a descriptive sense, particularly for MRE, Hausdorff distance, and IQR. These threshold violations indicate higher deviations and local variability for Pixel Photo, consistent with the modality’s reduced ability to satisfy clinical accuracy requirements.

In contrast, both the Pixel Video and the Sony-based reconstructions showed minimal threshold violations. Only 2 of 40 Pixel Video reconstructions and 1 of 40 Sony reconstructions exceeded the MRE threshold, and all other global metrics for both modalities remained within clinical limits. Descriptively, the Sony-based modality exhibited the lowest IQR and RMSE values, indicating reduced variability within the predefined clinical accuracy range. With respect to RMSE, it should be noted that the CT reference introduces an intrinsic spatial uncertainty of approximately 0.1 mm, corresponding to the voxel resolution, which represents a lower bound on measurable surface deviations. All reported RMSE values substantially exceed this bound, indicating that the observed variability reflects genuine reconstruction error rather than reference noise.

The MAE threshold of 4° was never violated. However, Sony-based reconstructions displayed slightly higher mean angular deviation and wider MAE distributions, suggesting the presence of fine-grained surface noise.

**Figure 7:**
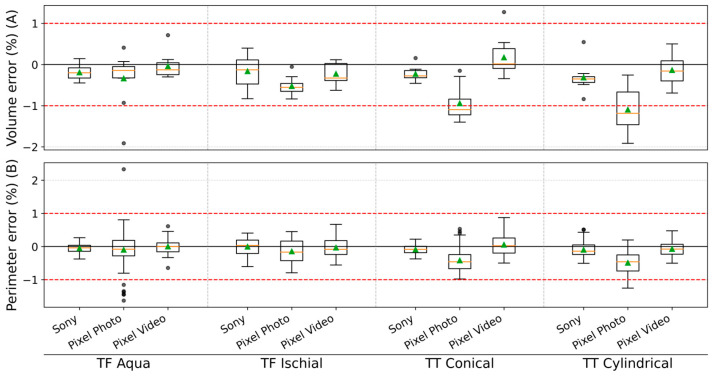
Photogrammetric reconstruction errors (%) across imaging modalities and limb models, in terms of (**A**) Volume and (**B**) perimeter. Box plots represent median values with interquartile ranges (IQR) across ten repeated acquisitions; green triangles indicate mean values. Red dashed lines denote clinically acceptable tolerance thresholds of ±1% for both volume and perimeter errors [35].

Consistent with these global metrics, both the Sony and smartphone video modalities remained well within the ±1% clinical limit for volume and perimeter errors (Figure 7). As detailed in Supplementary Tables S1 and S2, their bias and MDC values further support this stability. Only the Pixel Photo reconstruction slightly exceeded the 1% volume bias threshold. Nevertheless, MDC values for all modalities, including Pixel Photo, remained below the 3.5% limit. The corresponding absolute differences are reported in Supplementary Table S3: for Sony, volume differences ranged from −7.93 to −3.26 mL and perimeter differences from −0.33 to 0.05 mm; for Pixel Video, from −7.96 to 2.45 mL and −0.29 to 0.11 mm; and for Pixel Photo, from −22.33 to −13.41 mL and −1.84 to −0.49 mm, respectively.

Within the ROI, point density varied by acquisition modality. For the Pixel Photo reconstructions, the number of points ranged from 237,643 to 626,868, while Pixel Video reconstructions yielded 255,212 to 646,215 points. In contrast, reconstructions obtained with the Sony system exhibited substantially higher point densities, ranging from 1,069,659 to 2,245,215 points within the ROI.

### 3.3. Inter-Session Repeatability

We evaluated the repeatability of the reconstructed models under different acquisition conditions (Supplementary Table S4). Overall, the results indicate that smartphone video-based captures generally yielded slightly lower variability than smartphone photo-based reconstructions. For instance, TF Aqua reconstructed from Pixel Video achieved a pooled per-vertex SD of 0.14 mm and an MDC of 0.42 mm, compared to 0.33 mm and 1.02 mm, respectively, for the photo-based reconstruction. Similarly, the Sony-based reconstruction demonstrated the highest repeatability, with TT Conical showing the lowest SD (0.07 mm) and MDC (0.25 mm), reflecting consistent geometry across repetitions. Notably, for the Pixel Photo, the measurements obtained with the TF Aqua were unstable, whereas those with the TF Ischial were stable but exhibited a larger bias.

**Figure 8:**
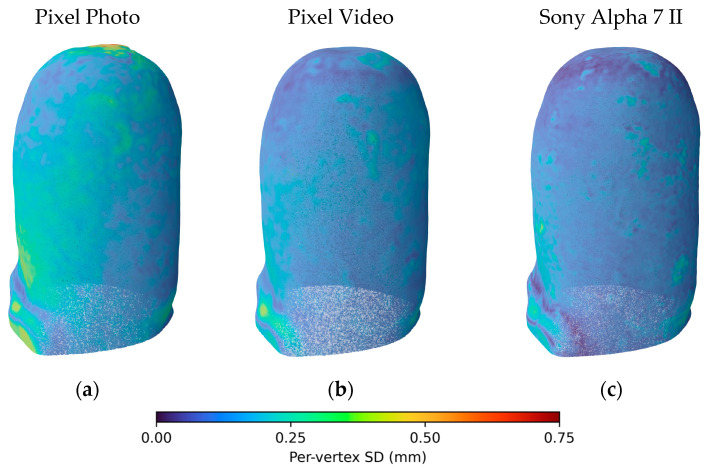
Visualization of inter-session local repeatability for the TT Cylindrical across three reconstruction types. Each panel shows a point cloud in which per-vertex standard deviation (SD) is encoded by color, with lower SD indicating higher local repeatability. From left to right: (**a**) Pixel Photo, (**b**) Pixel Video, and (**c**) Sony Alpha 7 II.

Figure 8 and Supplementary Figure S1 visualize spatial patterns of repeatability using per-vertex SD maps. For Pixel Photo, a distinct region of high variability is evident near the top of the model, suggesting inconsistent reconstruction of that area. This artifact is far less pronounced in Pixel Video and Sony-based reconstructions. Across all modalities, the largest SD values occur near edges, particularly the bottom-left border. These regions likely reflect the combined influence of smoothing, reduced feature coverage, and geometric ambiguity near object boundaries, common challenges in photogrammetry-based reconstructions.

### 3.4. Reproducibility

Across modalities, reproducibility of volume estimates showed no significant effect of limb model for the Sony (*p* = 0.689) or Pixel Video (*p* = 0.096). In contrast, Pixel Photo exhibited a significant effect of model shape (*p* = 0.0036). A similar pattern was observed for perimeter measurements, where Sony (*p* = 0.388) and Pixel Video (*p* = 0.335) again showed no detectable model influence, while Pixel Photo demonstrated a strong effect (*p* = 0.00026). These findings indicate that Pixel Photo is less reproducible across different limb geometries, whereas Pixel Video and Sony maintain stable performance independent of model shape.

## 4. Discussion

This study developed and validated a fully automated, low-cost photogrammetry pipeline for modeling residual limbs using a smartphone and a high-resolution full-frame camera. The workflow integrates frame selection, segmentation, metric scaling, and 3D reconstruction into a reproducible, script-driven process that requires no manual intervention. Accuracy was evaluated using 3D-printed phantom limbs and CT-derived reference meshes. For smartphone-video and full-frame camera inputs, the pipeline achieved sub-millimeter surface accuracy and relative volume and perimeter deviations within clinically accepted thresholds for prosthetic socket design.

### 4.1. Key Findings and Clinical Relevance

Our findings demonstrate that both Orthotics & Prosthetics–specific and global surface parameters fall within clinically acceptable thresholds for prosthetic applications when using either smartphone video or high-resolution inputs. The average errors observed in smartphone video-based and high-resolution-based reconstructions satisfied the criteria established by Cutti et al. [35], with |MRE| < 0.25 mm in 95% of cases for the video-based pipeline and 97.5% for the high-resolution pipeline, RMSE < 0.45 mm, MAE < 3°, and Hausdorff distance and IQR well below 1.8 mm and 0.4 mm, respectively. Importantly, these accuracy values should be interpreted relative to the intrinsic CT reference uncertainty, which defines a lower bound on resolvable surface deviations. Accordingly, reconstruction errors approaching this lower bound cannot be independently validated and should be interpreted as falling within the uncertainty of the CT reference itself, rather than as evidence of superior surface accuracy.

For VErel and perimeter error, the bias ranged from −0.32% to 0.17% and −0.10% to 0.05%, respectively, well under the 1% clinical threshold. This indicates high geometric and functional fidelity for these modalities [35], whereas the smartphone photo-based reconstruction showed larger deviations from clinically accepted thresholds. For Pixel Photo, volume bias ranged from −0.34% to −1.09% with MDC values between 0.63% and 1.80%, while perimeter bias ranged from −0.10% to −0.49% with MDC values between 0.90% and 1.41%, depending on socket geometry. Although MDC values remained below the 3.5% acceptability limit, only 62.5% of Pixel Photo measurements satisfied the |MRE| < 0.25 mm criterion, compared to ≥95% for the video-based and high-resolution pipelines [35].

The clinical implications of these accuracy levels are substantial, but must be interpreted in the context of established clinical tolerances. In this study, target accuracy thresholds were defined a priori based on previously published validation work for residual-limb scanning and prosthetic socket design, most notably by Cutti et al. [35]. These studies report that surface deviations on the order of sub-millimeter magnitude (|MRE| < 0.25 mm and RMSE < 1 mm) and VErel and relative perimeter errors within ±1% are clinically acceptable and sufficient to ensure reliable socket fit and shape reproduction. Within this framework, small deviations in residual limb geometry, particularly at pressure-sensitive areas such as the tibial tuberosity, fibular head, or ischial tuberosity, may cause discomfort, skin breakdown, or prosthesis rejection if not properly accounted for. Accurate and reliable surface representation is therefore essential for patient satisfaction and long-term prosthesis use [1]. At the same time, it is important to acknowledge that in vivo residual-limb geometry is influenced by soft-tissue deformation, posture, liner use, and donning and doffing procedures, all of which introduce variability beyond the measurement system itself [14]. Consequently, the sub-millimeter surface accuracy targeted in this work should be interpreted as a measurement-system performance goal that enables robust, repeatable digital representations, rather than as a claim that all clinically relevant tissue changes are directly measurable or actionable at that scale.

Although the present study did not evaluate performance across different stages of prosthetic monitoring, the results indicate that the proposed photogrammetry-based pipeline is suitable for use across multiple stages of residual limb management, not only during prosthetic socket fitting. In the acute postoperative phase (5–14 days), limb volume fluctuates substantially, with reported long-term reductions on the order of 17–35% [14]. In this phase, our method can enable accurate monitoring of swelling to determine when the limb has stabilized sufficiently for initial socket fitting [15]. Because of the high volumetric and perimeter reliability of our method, it can also be appropriate for longitudinal monitoring during the post-acute (4–8 weeks) and intermediate recovery (4–6 months) stages, and after limb maturation (≥12–18 months post-amputation), where clinically relevant diurnal volume fluctuations of approximately −1.5% to +2.0% have been reported [14,15]. In these periods, subtle shape and volume changes can inform timely socket adjustments as the limb continues to mature [15]. This broader applicability makes it particularly valuable for routine follow-up and potential remote care applications.

Beyond accuracy, the system’s low cost, short acquisition procedure (typically 2–10 min in our study), and minimal hardware requirements have important implications for accessibility. Unlike CT, MRI, or advanced scanning systems, our smartphone-compatible pipeline removes the need for specialized equipment or trained technicians [38]. This substantially broadens its potential use in rural clinics, mobile health units, and low-resource environments, thereby contributing to more equitable access to high-quality prosthetic care.

### 4.2. Measurement Repeatability and Reproducibility

Reproducibility across diverse limb geometries was a key strength of the system. Both smartphone video and high-resolution inputs performed consistently across all phantom shapes, including complex anatomies such as the TF ischial model, which features deep undercuts and pronounced curvature. This robustness is important for real-world clinical populations, where substantial anatomical diversity must be accommodated [8].

Repeatability results confirm that reconstructed models derived from video and high-resolution images exhibit minimal inter-session variation in clinically relevant metrics (RMSE, MRE, MAE). For Orthotics & Prosthetics characteristics, the MDC remained well below the 3.5% threshold recommended for clinical use. Consistent with global accuracy metrics, the photo-based pipeline performed worst across repeatability measures, while the high-resolution pipeline produced the lowest MDC values. Although the smartphone photo pipeline showed a slightly lower MDC in the TF ischial model, this result does not indicate superior performance, as it still exhibited the largest systematic bias among all methods. Vertex-level repeatability was highest for Sony (MDC 0.25–0.44 mm), followed by Pixel Video (0.36–0.43 mm) and Pixel Photo (0.35–1.02 mm). In prosthetic care, where monitoring subtle volumetric fluctuations is critical for adjusting socket fit, this level of repeatability provides strong support for clinical adoption [15].

As highlighted by Cutti et al. [35], reproducibility and inter-site consistency remain major barriers to adopting 3D scanning technologies in prosthetics. The strong reproducibility demonstrated here, particularly for smartphone video and high-resolution camera, therefore represents an important advancement toward clinical implementation.

### 4.3. Input Modality Performance: Video, Photo, Full-Frame

Clear performance differences emerged across input modalities. The smartphone photo-based pipeline consistently underperformed, exhibiting higher MRE, higher VErel, and lower repeatability across most models. These results suggest underlying issues in geometric consistency and metric calibration. Similar trends were observed by Teixeira Coelho et al. [23], who found that smartphone video-based photogrammetry generally produced smoother and more stable reconstructions than still photos.

Even though smartphone photo and video modes share nominal resolution and calibrated focal lengths, several factors likely contributed to poorer performance in still-image workflows. First, high dynamic range (HDR) photo processing often relies on multi-frame burst alignment and exposure fusion [39], which can introduce subtle geometric distortions that propagate into SfM. HDR video, on the other hand, typically uses per-frame temporal smoothing or dual-exposure sensors, producing frames with far less geometric distortion. Second, manual shutter activation introduces micro-movements, varying camera-to-object distance, and parallax errors, all detrimental to pose estimation. In contrast, video acquisition provides continuous viewpoint sampling and more uniform imaging geometry, leading to improved calibration and reduced reconstruction noise.

Among the high-performing modalities, the Sony camera produced the greatest overall accuracy and repeatability, although slightly elevated MAE values suggest localized surface noise, which may be partially attributable to higher ISO settings and the resulting image noise, as well as to higher image sharpness and richer texture detail, a pattern also noted by Teixeira Coelho et al. [23]. In addition, the substantially higher point density achieved with the Sony reconstructions can amplify the impact of small-scale noise on normal estimation, leading to larger normal deviations compared with the Pixel Video and Photo modalities. Furthermore, the Sony A7 II employs in-body mechanical sensor stabilization, in which the image sensor physically moves during acquisition. While beneficial for reducing motion blur, this mechanism implies that camera intrinsic parameters are not strictly constant across frames, potentially limiting the maximum achievable reconstruction accuracy in SfM pipelines that assume fixed intrinsics. Meanwhile, smartphone video achieved results within clinical thresholds for 95% of scans and matched or exceeded Sony performance on several metrics. Considering cost, acquisition speed, and hardware availability, smartphone video represents a highly practical and clinically viable acquisition modality.

### 4.4. Comparison with Previous Studies

The accuracy demonstrated here aligns with, and in several respects exceeds, that of established 3D modeling systems. Structured-light scanners, commonly used in prosthetics, report relative volume deviation SDs of 1.78–2.04% [13], whereas our smartphone video–based photogrammetry achieved substantially lower variability. Although structured-light results were obtained on real residual limbs, whereas our evaluation used phantoms, the comparison highlights the strong potential of photogrammetry for reliable clinical use.

When comparing our findings with those of Cutti et al. [35], who evaluated structured-light scanners on the same phantom models, our smartphone video-based approach achieved comparable accuracy. Structured-light scanning yielded a maximum RMSE of 0.27 mm and MRE between −0.05 mm and 0.05 mm, slightly lower than our photogrammetry results, while our MAE remained below 2°, outperforming the structured-light system (1–2.5°). Perimeter bias and volume bias were likewise comparable and within clinical limits. Squibb et al. [16] reported similarly precise laser-based 3D scanning measurements for transtibial limbs. However, both structured-light and laser scanning systems are costly and demand extensive operator training, which limits their scalability compared to our smartphone-based pipeline.

Our results extend prior photogrammetry research by addressing gaps in automation, scaling, and ground-truth validation. Earlier studies [20,21,22,24] provided promising feasibility evidence but relied on manual workflows, commercial software, or lacked high-accuracy validation references, and reported residual errors that remain above commonly accepted clinical thresholds (e.g., Walters et al. [22] reported volume differences of −2.9% and −1.0% and surface RMSE of 1.99–2.36 mm). In contrast, our pipeline is fully automated, includes robust metric scaling, and validates reconstructions against CT-derived ground-truth models, providing quantitative evidence of clinical feasibility. Compared with CT or MRI, which are accurate but impractical for routine external shape capture [11], our method offers a compelling balance between accuracy, cost, and operational simplicity.

### 4.5. Scalability and Automation

The photogrammetry pipeline is implemented as a fully automated, modular workflow orchestrated via Bash scripting and open-source tools. By eliminating manual intervention, the method reduces inter-operator variability, a persistent challenge in plaster casting and handheld scanning [9,11]. The pipeline’s standardized structure also enables reproducible measurement in multicenter studies and clinical trials.

Scalability is further enhanced by the use of standard smartphones and inexpensive hardware, enabling adoption in low-resource settings, community rehabilitation centers, and remote environments [20,24,35]. Its automation and device independence also facilitate integration with digital prosthetic workflows, including remote care and AI-driven fit prediction [18,24].

### 4.6. Technical Limitations and Assumptions

While demonstrating strong accuracy, repeatability, and reproducibility in controlled settings, several limitations should be acknowledged. First, the current processing time, approximately 1 h and 30 min per reconstruction, represents a substantial barrier to routine point-of-care clinical use. This runtime is considerably longer than that of commercially available structured-light scanners or smartphone-native scanning applications, which can provide near–real-time visual feedback and rapidly export models for downstream fabrication [22,35]. In the present work, processing time was not optimized, as the pipeline prioritized automation, reproducibility, and transparency using open-source components rather than clinical turnaround speed. Future optimization steps include reducing image and frame counts, tuning reconstruction parameters, leveraging GPU acceleration for SfM and MVS, and exploring off-device or cloud-based processing to better align with clinical workflow requirements.

Second, validation was performed using rigid, 3D-printed phantoms. Although ideal for controlled CT benchmarking, these lack key biological characteristics such as soft tissue deformability, skin heterogeneity, hair, and reflective properties. In particular, the surface texture of the phantoms does not replicate the spatial and photometric complexity of real human skin, whereas SfM fundamentally relies on sufficient and well-distributed surface features for stable correspondence estimation. These factors may influence photogrammetric performance in vivo, particularly in areas prone to deformation or variable texture [35]. In addition, the dense distribution of calibration markers used in this study was specifically designed for phantom-based validation to ensure accurate scale recovery and is not intended to be directly replicated on patients. In a clinical context, attaching large numbers of discrete markers to the residual limb would be impractical. However, several clinically feasible alternatives exist, including integrating scale references into a detachable support or base, using a reusable external calibration frame, incorporating printed fiducials into a liner or sleeve, or reducing the marker count while preserving sufficient geometric constraints for scale estimation. These strategies provide a clear pathway for translating the proposed pipeline to in vivo use without imposing excessive burden on patients or clinicians.

Third, all models were scanned in a static, unloaded condition. Residual limb shape is posture-dependent; small changes in orientation or weight-bearing can alter local volume, affecting socket fit. Real-world scanning conditions also involve compressive liners and dynamic musculature, which were not represented here [15]. Moreover, the approximately two-minute acquisition duration makes the procedure potentially sensitive to subject motion, which may introduce reconstruction artifacts in live subjects. However, moderate global movement of the limb may be accommodated in photogrammetry-based reconstruction, provided that the region of interest can be reliably segmented across views. Under this condition, photogrammetry may be less sensitive to global translational or rotational motion. Evaluating reconstruction fidelity in live subjects, under both unloaded and functional postures, is a critical next step.

Fourth, lighting conditions strongly affect photogrammetry [40,41]. Although the pipeline includes sharpness-based frame selection, exposure variability was managed manually. A dedicated scanning app that provides real-time exposure control could minimize this source of variability [42]. In this study, acquisitions were performed outdoors under natural daylight to promote relatively uniform, diffuse illumination and to avoid hard shadows or flicker artifacts associated with artificial lighting. While outdoor daylight enabled uniform illumination, reliance on uncontrolled natural lighting may limit reproducibility in some real-world settings and represents a potential constraint for future clinical deployment beyond the scope of the present validation.

Fifth, metric scaling in the proposed pipeline is based on the physical dimensions of printed ArUco markers [29]. All markers used in this study were produced using the same laser printer, and their dimensions were manually verified prior to data acquisition to ensure consistency. Absolute scale accuracy nevertheless remains ultimately tied to the fidelity of the printing and verification process. While this does not impact the validity of the present results, future clinical or metrologically traceable deployments may benefit from alternative scaling strategies, such as factory-calibrated fiducials, certified reference objects, or integrated calibration frames, to ensure standardized absolute accuracy and formal traceability.

Finally, while the pipeline minimizes operator influence, user skill and environmental conditions may still affect reconstruction quality [20,35]. Future improvements such as guided acquisition interfaces, real-time quality feedback, and automated camera parameter adjustment would significantly improve usability and robustness in remote and resource-limited settings [24,42].

## 5. Conclusions

This study presents a fully automated, low-cost photogrammetry pipeline capable of generating accurate and clinically meaningful 3D models of residual limbs using only a smartphone or a consumer-grade digital camera. By integrating adaptive frame selection, deep learning–based segmentation, robust metric scaling with ArUco markers, and standardized SfM–MVS reconstruction, the workflow eliminates manual intervention and operator dependency, two major barriers to widespread clinical adoption of digital limb modeling.

Validation against CT-derived ground-truth meshes demonstrated that both smartphone video and high-resolution inputs achieve sub-millimeter surface accuracy, perimeter and volume biases well within ±1%, and high repeatability across diverse limb geometries. These performance levels satisfy established clinical requirements for prosthetic socket design and monitoring of residual limb maturation. Among all modalities tested, smartphone video provided the best trade-off between accuracy, acquisition speed, and accessibility, making it a strong candidate for integration into routine prosthetic workflows.

The scalability and hardware independence of the pipeline enable deployment across a range of clinical environments, including rural, mobile, and resource-limited settings. While the evaluation relied on rigid phantoms and controlled acquisition conditions, the results establish a solid foundation for future in vivo studies. Examining performance on live subjects, under both unloaded and functional postures, will be an important next step toward clinical translation.

Overall, the proposed photogrammetry-based pipeline shows promising results under controlled, phantom-based validation conditions and may provide an alternative approach for residual limb modeling. However, clinical applicability will require further steps, including in vivo validation under diverse conditions, reduction in processing time to meet clinical workflow constraints, and the development of guided acquisition tools to ensure robust and practical use.

## Figures and Tables

**Table 1:** Summary of image and video acquisition parameters used during photogrammetric capture of residual limb models. Parameters are listed for both camera modalities (Google Pixel 6a and Sony Alpha 7 II).

Parameter	Google Pixel 6a	Sony Alpha 7 II	Notes
Camera type	Smartphone (rear camera)	Full-frame mirrorless	-
Resolution	3840 × 2160 pixels (8.29 MP)	6000 × 4000 pixels (24 MP)	-
Focal length	27.9 mm	50 mm	Full-frame equivalent
Aperture	f/1.7	f/14	-
Frame rate	30 fps	N/A	Recorded videos
Shutter speed	Automatic (1/500–1/2000 s)	Fixed (1/500 s)	Controls motion blur
ISO	Automatic (50–100)	Automatic (800–3600)	Controls light sensitivity
Focus	Manual	Manual	Fixed for all models
Camera distance to object	~50 cm	~80 cm	Maintained manually
Lighting conditions	Outdoor daylight	Outdoor daylight	-

## Data Availability

The raw data supporting the conclusions of this article will be made available by the authors on request.

## Supplementary Materials

The following supporting information can be downloaded at: https://www.mdpi.com/article/10.3390/s26041251/s1, Table S1: Bias and minimal detectable change (MDC) for volume measurements across imaging modalities and limb geometries; Table S2: Bias and minimal detectable change (MDC) for perimeter measurements across imaging modalities and limb geometries; Table S3: Absolute differences in volume (mL) and perimeter (mm) between photogrammetric reconstructions and CT-derived ground-truth models; Table S4: Repeatability of reconstructed residual limb models across imaging modalities and acquisition conditions; Figure S1: Visualization of inter-session local repeatability across three reconstruction types for the following models. The supporting material can be downloaded at: https://doi.org/10.5281/zenodo.18632965.

## Abbreviations

The following abbreviations are used in this manuscript:

CT	Computed Tomography
MRI	Magnetic Resonance Imaging
SfM	Structure-from-Motion
MVS	Multi-View Stereo
ArUco	Augmented Reality University of Cordoba
TF	Transfemoral
TT	Transtibial
ROI	Region of interest
MPT	Mid-Patellar Tendon
BAR	Midpoint between the origin of the Adductor Longus and the Ischial Ramus
MRE	Mean radial error
RMSE	Root mean square error
IQR	Interquartile range
MAE	Mean angular error
MDC	Minimal detectable change
SD	Standard deviation
P95	95th percentile
VErel	Relative volume error
HDR	High dynamic range
