# Supplementary material for: Smartphone-Based Automated Photogrammetry for Reconstruction of Residual Limb Models in Prosthetic Design
Source: Sensors (Basel). 2026 Feb 14;26(4):1251. doi: 10.3390/s26041251 (PMC12944613; doi:10.3390/s26041251)
Supplement: Supplementary file 1 [file sensors-26-01251-s001.zip › sensors-4066159-supplementary.pdf]

Supplementary Methods

Overview of the automated photogrammetry pipeline

The entire photogrammetry workflow is executed through a fully automated script that sequentially performs:

1. extraction and selection of high-quality frames,
2. background removal,
3. metric scaling using ArUco markers,
4. structure-from-motion (SfM),
5. multi-view stereo (MVS) densification,
6. smoothing.

No manual post-processing, mesh editing, or commercial software is used at any stage. All steps rely on open-source tools (Python, COLMAP, OpenMVS, PyMeshLab).

1. Extraction and selection of high-quality frames

To standardize input for photogrammetric reconstruction, preprocessing is implemented to extract and prepare images from both still photographs and video recordings. For video-based input, frames are extracted using FFmpeg, and a sharpness metric is computed for each frame based on the variance of the Laplacian. To select the most informative frames, an adaptive sharpness-based selection algorithm is applied. A moving window (size = 5 frames) is used to compute a local median sharpness and interquartile range (IQR), capturing local variability in image quality. An adaptive threshold (T) is then defined for the i-th frame as:

$$T_i = Median_i + IQR_i/4 \quad (1)$$

Frames exceeding this threshold are retained, ensuring only those with above-average local sharpness are selected. This approach is specifically designed to minimize the inclusion of frames affected by motion blur during handheld video recording.

All selected images are renamed in a consistent format and corrected for orientation using EXIF metadata, ensuring that only top-left-aligned images are retained.

2. Background removal

To isolate the object of interest, background removal is performed using CarveKit (v4.1.2) (Selin, 2024) with model TRACER-B7 (Lee et al., 2022) and Compute Unified Device Architecture (CUDA) backend. The resulting alpha masks are converted to binary masks by thresholding the transparency channel at 50%, producing white foreground and black background regions.

3. Metric scaling using ArUco markers

Augmented Reality University of Cordoba (ArUco) markers are used to compute the physical scale and recover the extrinsic camera parameters for each frame. Given a set of images, known tag side length and IDs, and intrinsic camera parameters, the process detects the 2D positions of the tags, estimates their 3D poses, and jointly optimizes both tag and camera positions into a consistent map. The result is a set of scaled extrinsic camera parameters in a shared coordinate frame and corresponding tag poses. This is implemented using an adapted version of the open-source pytagmapper library (MIT license) (Liu, 2021), which is extended to improve robustness and integrated into the photogrammetry pipeline via additional custom scripts.

Tag detection is performed using a modified version of pytagmapper's detection script, which internally uses OpenCV's ArUco module. The adapted version adds support for filtering specific tag IDs via command-line input and applies sub-pixel refinement (cv2.cornerSubPix) to improve localization accuracy. Detected tags are saved per image in individual text files for further processing.

Camera and marker poses are estimated using a two-stage Perspective-n-Point (PnP) pipeline. For each image, the 2D corner positions of visible markers are matched to their known 3D coordinates based on tag size. An initial camera pose is computed using cv2.solvePnPRansac, which estimates the 3D transformation while rejecting outlier correspondences via Random Sample Consensus (RANSAC). This initial estimate is then refined using cv2.solvePnPRefineLM, which minimizes the reprojection error through Levenberg–Marquardt optimization. Compared to the original implementation, which uses only linear PnP, this approach is more robust and provides more accurate pose estimates. To ensure geometric consistency, a new camera is only added to the map if it has at least 15 overlapping tags with the existing tag set. After each addition, all camera and tag poses are jointly refined using a message-passing–based optimizer. This method iteratively linearizes the reprojection error around the current pose estimates, computes updates using the Jacobians of the error function, and applies these updates to all variables in the graph. The process continues until the average reprojection error converges, similar in principle to nonlinear least-squares solvers used in factor graph optimization.

To integrate the result with the SfM pipeline, a custom script is added to convert the extrinsic camera parameters into a compatible camera trajectory. For each camera, the translation components of the 4×4 transformation matrix are extracted and saved in text format. This allows initial alignment of the SfM reconstruction to the ArUco-based reference frame.

The entire workflow is automated using a Bash script that prompts the user to select the appropriate intrinsic camera parameters, specify the physical tag size, and enter valid marker IDs. It runs each processing stage in sequence and includes a retry mechanism for the mapping phase. If solvePnPRansac fails due to poor initialization, the script automatically restarts the process. This setup ensures reproducibility and robustness for large image datasets.

4. Structure-from-motion (SfM)

Structure-from-motion (SfM) is performed using COLMAP (v3.11.1) (Schönberger, 2024; Schönberger & Frahm, 2016), an open-source photogrammetry pipeline licensed under the BSD license (ETH Zurich and UNC Chapel Hill). All steps are executed inside a Docker container to ensure consistent runtime environments and enable GPU acceleration. The reconstruction pipeline consists of feature extraction, exhaustive matching, incremental mapping, and alignment.

During SfM feature extraction, SIFT keypoints and descriptors are computed using GPU acceleration. A single calibrated camera model (PINHOLE) is assumed for all images, and the binary masks made with CarveKit are applied to restrict feature detection to relevant regions. The intrinsic camera parameters are specified and fixed throughout the pipeline. These parameters are identical to those used for estimating the scale factor from ArUco marker detections, ensuring consistency between metric scaling and the reconstructed scene geometry.

Exhaustive matching is used to establish correspondences between all image pairs. To ensure robustness, the script automatically detects known runtime issues and re-executes the matcher if necessary. Sparse reconstruction is then performed using COLMAP's incremental mapper. During this process, bundle adjustment is used to jointly optimize camera poses and selected intrinsic parameters by minimizing the global reprojection error. The focal length and principal point are kept fixed to match the external calibration, while radial distortion parameters are refined to compensate for minor model inaccuracies. If the mapping process fails due to repeated initialization attempts, the script extracts the best initial image pair from the logs and restarts the process using this specific pair to initialize the reconstruction.

Following sparse reconstruction, the images are undistorted based on the estimated intrinsic camera parameters to prepare for dense reconstruction. The resulting model is then aligned to a predefined coordinate system using the reference camera positions extracted in the ArUco pipeline. This alignment is performed using COLMAP's built-in rigid alignment tool. Finally, the model is exported in COLMAP's text format to facilitate dense reconstruction.

5. Multi-view stereo (MVS) densification

Multi-view stereo (MVS) is performed using the OpenMVS library (v2.3.0) (Cernea, 2020) licensed under AGPL-3.0 (cDc Seacave) within a CUDA-enabled Docker container. The input to this stage is the sparse, undistorted COLMAP reconstruction aligned to a common coordinate frame. OpenMVS is used to generate a dense point cloud, surface mesh, and textured model.

The COLMAP model is first converted to the OpenMVS internal format using InterfaceCOLMAP, which imports the sparse structure, camera poses, and image data. The binary mask made with CarveKit is provided to restrict densification to the object of interest. Dense stereo reconstruction is then performed using DensifyPointCloud, producing a high-resolution point cloud. To limit processing time, the high-resolution Sony camera images were downscaled once during densification. A visibility filter was subsequently applied to the point cloud, retaining only points with sufficient visibility. Surface reconstruction is achieved using

ReconstructMesh, which estimates a watertight surface from the dense points, resulting in a dense 3D model.

6. Smoothing

To smooth the reconstructed models, PyMeshLab (v2023.12) (Alessandro Muntoni et al., 2025) licenced under the GPL License was used to perform a surface-preserving Laplacian smoothing filter. This algorithm iteratively moves each vertex toward the average position of its neighboring vertices, provided that the new position remains approximately on the original surface. A maximum normal angle displacement of 90° was applied, with a total of 20 iterations performed.

Validation

1. Limb model preparation

Four different types of lower-limb amputation models were selected based on the classification by Cutti et al. (2024). These include:

- TF Aqua: A transfemoral socket for a long residual limb, also featuring ischial containment but without any undercuts or indentations around the proximal brim.
- TF Ischial: A transfemoral socket for a long residual limb, incorporating ischial containment with undercuts and indentations around the proximal brim.
- TT Conical: A transtibial socket for a long residual limb, with a patellar tendon bearing design and a conical shape.
- TT Cylindrical: A transtibial socket for a mid-length residual limb, with a total surface bearing design and cylindrical geometry.

These digital 3D models were obtained from the Dryad data repository, shared by Cutti et al. (2024) as part of their experimental dataset. The meshes were originally generated using the metrological-grade handheld scanner EINScan FreeScan 5X (SHINING 3D Tech. Co., Ltd., Hangzhou, China), which served as the gold standard in the referenced study.

To prepare the models for 3D printing, a flat stand was added to each socket, and any remaining holes in the mesh were closed using Blender. The models were then fabricated in white SLA resin via MakerVerse (Berlin, Germany). After printing, the models were spray-painted in a base skin tone, with additional brush strokes in various skin-like colors to simulate realistic surface variations and support photogrammetric feature detection.

To enable scaling, thirty-two 5×5 ArUco markers (2 cm) were affixed to the stand of the models. To further enhance feature extraction, additional black lines were drawn on the stand.

2. Ground-truth generation

The 3D-printed models were scanned on a TESCAN UniTOM XL and reconstructed in Penthera™ with isotropic voxel spacing of 100 µm. A custom Python pipeline processes the volume in slabs of 500 slices (≈50 mm). Each slab includes a ±2.0 mm halo, a small overlap added above and below the slab to keep edge context and avoid boundary artifacts. The slices of the stand were excluded. A single global threshold is computed once with Otsu's method (automatic, data-driven thresholding) from a subsample of the slices, and that fixed threshold is used for all slabs. The exterior air is then identified by binary propagation seeded at the slab borders (flood-fill from the outside), and interior air is filled, leaving only the object's outer shell. From this shell we compute a signed distance field (SDF) (a grid where values are positive inside, negative outside, zero on the surface), apply a light Gaussian smoothing (σ = 0.5 vox), and extract the zero level set with marching cubes (standard surface polygonization) while preserving the native 0.10 mm spacing. This reduces voxel stair-stepping effect. Only faces whose centroids fall inside the slab core are retained to prevent duplication between slabs.

After concatenating the per-slab meshes, we applied ε-welding: vertex coordinates were rounded to the nearest 0.025 mm (¼ of the 0.10 mm voxel), and coincident vertices were merged, which removes hairline seams at slab boundaries while altering geometry by at most 0.025 mm. After mesh cleanup (removing duplicate/degenerate faces) Taubin smoothing is applied (3 iterations; $\lambda = 0.33$, $\nu = -0.34$), a non-shrinking filter that reduces voxelization without inflating volume, orient normals outward, and export the triangular mesh for analysis. The same surface-preserving smoothing technique as employed for the photogrammetry reconstructions was applied, using a maximum normal angle displacement of 90° and 20 iterations.

3. Image acquisition setup

To assess both precision and repeatability of each photogrammetry system, the acquisition protocol was repeated ten times for each limb model and camera configuration, for a total number of one hundred twenty acquisitions.

Image and video data were acquired using two cameras: the high-resolution setup employed a Sony Alpha 7 II full-frame mirrorless camera (24.3 MP, 6000×4000 pixels), equipped with a Sony FE 50 mm f/2.5 G prime lens. Images were captured at f/14 aperture with a shutter speed of 1/500 s, and automatic ISO enabled to ensure consistent exposure across frames. The secondary device was a Google Pixel 6a smartphone, which captured 3840×2160 pixel images and videos at 30 frames per second using its native f/1.7 wide-angle lens (27.9 mm full-frame equivalent). The OpenCamera app was used for data acquisition. For both systems, manual focus was used and kept constant for each model.

All image acquisitions were conducted outdoors under natural daylight conditions to minimize motion blur. The models were photographed in a circular pattern from three distinct perspectives: low-angle (worm's-eye), eye-level frontal, and high-angle (bird's-eye) views. The angular displacement between consecutive images ranged from 1° to 15°, and efforts were made to ensure that the entire model was fully captured within each image.

Intrinsic camera parameters were estimated using a custom Python script based on OpenCV's calibration routines. For each camera, 50 images of a printed checkerboard target (11×8 squares, 10×7 inner corners, 2.5 cm square size) were acquired at varying angles and distances. Checkerboard corners were detected using subpixel refinement. The intrinsic matrix and distortion coefficients were estimated by minimizing the reprojection error via nonlinear least-squares bundle adjustment (cv2.calibrateCameraExtended). Object points were automatically scaled based on the known square size, and strict convergence criteria were applied to ensure subpixel accuracy. The final calibration output included the intrinsic matrix, distortion coefficients, per-view reprojection errors, and the overall root-mean-square error (RMSE), used as the calibration quality metric.

4. Performance assessment

Below, the validation protocol used to assess accuracy and repeatability of the 3D models against CT within a clinically defined region of interest (ROI) is described. The accuracy of each reconstruction was assessed using both global deviation metrics and clinical shape descriptors, as described by Cutti et al. (2024).

The ROI is anatomically bounded from the most distal point of the limb model to, for transtibial (TT) limbs, the Mid-Patellar Tendon (MPT), and for transfemoral (TF) limbs, the midpoint between the origin of Adductor longus and the ischial ramus (BAR). Operationally, this ROI is implemented as an axis-aligned 3D box defined in the CT reference frame; all subsequent processing, measurements, and statistics are restricted to geometry inside this ROI.

To place models in a common anatomical frame without introducing scale or shear, rigid-only registration (restricted to the CT ROI as the registration driver. After estimating normals where needed, we first run a coarse alignment using Fast Point Feature Histograms (FPFH) feature matching with RANSAC (point-to-point), followed by multi-scale point-to-plane iterative closest point (ICP) refinement (Rusu et al., 2009). The resulting transform is applied to the test model so that both datasets are expressed in the CT frame. Both point clouds are then cropped to the ROI.

The signed radial error is calculated by mapping every CT (target) point to its nearest modelled (source) point and assigning the distance sign by the local source normal (positive along the outward source normal). From these signed distances, we report the mean radial error (MRE), interquartile range (IQR), the root mean squared radial error (RMSE), and the Hausdorff distance (max |error|) (Cutti et al., 2024). In addition, normal angle errors are computed by comparing each target normal to its nearest source normal. In this analysis the mean angel error (MAE) is reported. Based on previous clinical studies by Sanders et al., (2012) and Seminati et al., (2017), and in accordance with the criteria established by Cutti et al., (2024), the MRE must not exceed 0.25 mm, the IQR must be smaller or equal to 0.4 mm the MAE must remain below 4°, the Hausdorff distance must not surpass 1.8 mm, and the RMSE must stay under 1 mm in order to ensure no clinically relevant effect on prosthesis–socket agreement.

Orthotics & Prosthetics (O&P) cross-sectional metrics are derived by partitioning the ROI into ten evenly spaced axial slices along the CT z-axis (Cutti et al., 2024). For each slice, the cross-section perimeter is computed. For volumetric analysis, both triangle meshes are cropped to

the region of interest (ROI), after which the absolute volume within the ROI is computed and compared against the ground truth. The accuracy of these O&P metrics is evaluated by quantifying the bias and the repeatability across repetitions using the minimal detectable change (MDC). To prevent clinically appreciable differences in fit, gait, and comfort, both volume and perimeter must demonstrate a bias of less than 1% and an MDC below 3.5%. (Cutti et al., 2024; Dickinson et al., 2016; Mehmood et al., 2019). To assess reproducibility across the different models for each reconstruction type, a one-way ANOVA was performed on the VE_{rel} and the relative perimeter error averaged over sections with amputation type as the factor and a significance level of $\alpha = 0.05$.

Repeatability was further evaluated using ten repeated point clouds of the same limb segment. Each repeat was rigidly aligned to the CT frame using the same ROI-anchored registration pipeline and subsequently cropped to the predefined ROI. Within this repeat set, a medoid point cloud (i.e., the cloud minimizing the sum of pairwise symmetric Chamfer distances to all others) was selected to act as an internal reference. For every repeat, signed point-to-surface distances to the medoid were computed at all sampled vertices. These signed distance fields were used to evaluate both local and global repeatability across the repetitions.

For each medoid vertex, the standard deviation of its signed distances across repeats was computed. This per-vertex SD quantifies local repeatability, i.e., the random measurement variability at the same anatomical location. These local dispersions (and associated percentiles) were visualized as color maps to identify regions exhibiting higher variability.

To summarize repeatability in a single metric, all signed distances from all vertices and all repeats were pooled into one distribution. The standard deviation of this full distribution reflects both local random variation and systematic spatial differences between repeats and the medoid (e.g., small residual alignment offsets or consistent regional shape deviations). A global repeatability metric was obtained via the minimum detectable change (MDC) at the 95% confidence level, defined as:

$$MDC = 1.96 * \sqrt{2} * SD_{global} \quad (2)$$

The MDC value expresses that two measurements of a randomly selected point within the same anatomical region are expected to differ by no more than the MDC in 95% of repeated acquisitions.

This combination of rigid alignment and clinically meaningful error metrics allows for a comprehensive assessment of the photogrammetry pipeline's geometric fidelity, consistent with best practices for validation in prosthetic applications (Cutti et al., 2024).

Supplementary Figures and Tables

Supplementary Table S1: Bias and minimal detectable change (MDC) for volume measurements across imaging modalities and limb geometries. Bias represents the mean signed difference (%) between photogrammetric reconstructions and CT-derived ground-truth models, while MDC reflects the smallest detectable percentage change exceeding measurement variability. Values are expressed as mean ± standard deviation (SD) from ten repeated acquisitions. The clinical acceptability threshold of ±1% (see Figure 10) is indicated for reference.

	Pixel Photo (%)		Pixel Video (%)		Sony Alpha 7 II (%)	
	**Bias**	**MDC**	**Bias**	**MDC**	**Bias**	**MDC**
TF Aqua	-0.34±0.65	1.80	-0.04±0.30	0.84	-0.19±0.20	0.54
TF Ischial	-0.53±0.23	0.63	-0.23±0.26	0.71	-0.16±0.41	1.14
TT Conical	-0.94±0.42	1.16	0.17±0.48	1.32	-0.23±0.17	0.48
TT Cylindrical	-1.09±0.52	1.45	-0.14±0.36	1.00	-0.32±0.35	0.96

Supplementary Table S2: Bias and minimal detectable change (MDC) for perimeter measurements across imaging modalities and limb geometries. Bias denotes the mean signed difference (%) between photogrammetric reconstructions and CT-derived ground-truth models, and MDC indicates the smallest measurable change exceeding intra-session variability. Values are expressed as mean ± standard deviation (SD) from ten repeated acquisitions. The clinical acceptability threshold of ±1% (see Figure 11) is indicated for reference.

	Pixel Photo (%)		Pixel Video (%)		Sony Alpha 7 II (%)	
	**Bias**	**MDC**	**Bias**	**MDC**	**Bias**	**MDC**
TF Aqua	-0.10±0.51	1.41	-1.2E-4±0.21	0.57	-0.05±0.15	0.40
TF Ischial	-0.15±0.33	0.92	-0.03±0.28	0.79	-3.7E-3±0.24	0.70
TT Conical	-0.42±0.35	0.96	0.05±0.31	0.87	-0.09±0.13	0.36
TT Cylindrical	-0.49±0.32	0.90	-0.07±0.21	0.59	-0.10±0.22	0.61

Supplementary Table S3: Absolute differences in volume (ml) and perimeter (mm) between photogrammetric reconstructions and CT-derived ground-truth models. Values are reported as mean ± standard deviation (SD) over ten repeated acquisitions.

	Pixel Photo		Pixel Video		Sony	
	**Volume (ml)**	**Perimeter (mm)**	**Volume (ml)**	**Perimeter (mm)**	**Volume (ml)**	**Perimeter (mm)**
TF Aqua	-13.95±26.74	-0.49±1.97	-1.68±12.46	0.01±0.85	-7.93±8.03	-0.21±1.97
TF Ischial	-18.37±7.89	-0.60±1.29	-7.96±9.00	-0.18±1.03	-5.68±14.42	0.05±0.90
TT Conical	-13.41±5.96	-1.34±1.06	2.45±6.80	0.11±0.96	-3.26±2.45	-0.25±0.39
TT Cylindrical	-22.33±10.66	-1.84±1.25	-2.79±7.38	-0.29±0.80	-6.45±7.08	-0.33±0.81

Supplementary Table S4: Repeatability of reconstructed residual limb models across imaging modalities and acquisition conditions. The table reports the pooled per-vertex SD, IQR, P95, and the global MDC of surface deviation metrics obtained from ten repeated acquisitions for each limb model and device. Lower SD and MDC values indicate higher measurement repeatability.

			**Local (pooled)**		**Global**
		**SD (mm)**	**IQR (mm)**	**P95 (mm)**	**MDC (mm)**
TF Aqua	**Pixel Photo**	0.33	0.22	0.35	1.02
	**Pixel Video**	0.14	0.14	0.22	0.42
	**Sony Alpha 7 II**	0.09	0.12	0.11	0.29
TF Ischial	**Pixel Photo**	0.11	0.12	0.14	0.35
	**Pixel Video**	0.11	0.15	0.19	0.36
	**Sony Alpha 7 II**	0.14	0.20	0.18	0.44
TT Conical	**Pixel Photo**	0.13	0.14	0.18	0.42
	**Pixel Video**	0.13	0.13	0.18	0.38
	**Sony Alpha 7 II**	0.07	0.08	0.10	0.25
TT Cylindrical	**Pixel Photo**	0.19	0.25	0.27	0.61
	**Pixel Video**	0.13	0.16	0.19	0.43
	**Sony Alpha 7 II**	0.11	0.09	0.18	0.36

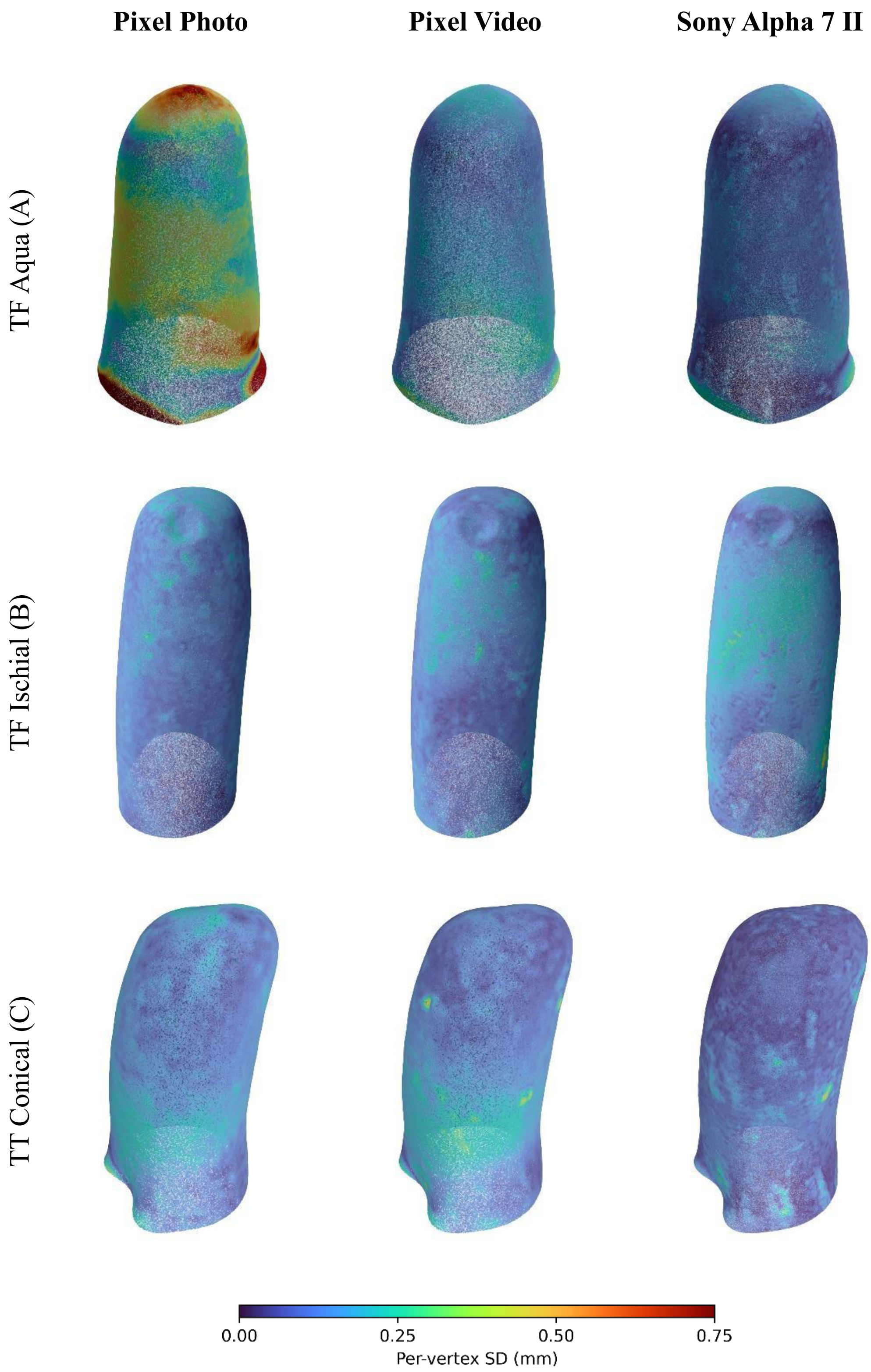

Supplementary Figure S1: Visualization of inter-session local repeatability across three reconstruction types for the following models: *A) TF Aqua; B) TF Ischial; C) TT Conical*. Each panel shows a point cloud in which per-vertex standard deviation (SD) is encoded by color, with lower SD indicating higher local repeatability. From left to right: Pixel Photo, Pixel Video, and Sony.
